# Co-occurrence of depressive, anxiety, and somatic symptoms: trajectories from adolescence to midlife using group-based joint trajectory analysis

**DOI:** 10.1186/s12888-019-2203-7

**Published:** 2019-08-01

**Authors:** Tea Lallukka, Gashaw B. Mekuria, Tapio Nummi, Pekka Virtanen, Marianna Virtanen, Anne Hammarström

**Affiliations:** 1 Department of Public Health, University of Helsinki, Helsinki, Finland; 2 Finnish Institute of Occupational Health, Helsinki, Finland; 3 University of Tampere, Faculty of Social Sciences, Tampere, Finland; 4 University of Tampere, School of Natural Sciences, Tampere, Finland; 5 School of Educational Sciences and Psychology, University of Eastern Finland, Joensuu, Finland; 6 Department of Epidemiology and Global Health, Umeå University, Umeå, Sweden; 7 Stress Research Institute, Stockholm University, Sweden and Institute of Environmental Medicine, Karolinska Institutet, Stockholm, Sweden

**Keywords:** Mental health, Epidemiology, Adolescents, Adults

## Abstract

**Background:**

Co-occurrence of mental and somatic symptoms is common, and recent longitudinal studies have identified single trajectories of these symptoms, but it is poorly known whether the symptom trajectories can also co-occur and change across the lifespan. We aimed to examine co-occurring symptoms and their joint trajectories from adolescence to midlife.

**Methods:**

Longitudinal data were derived from Northern Sweden, where 506 girls and 577 boys aged 16 years participated at baseline in 1981 (99.7% of those initially invited), and have been followed up in four waves until the age of 43. Survey data were collected about depressive, anxiety, and somatic symptoms. Potential joint development of this three-component symptom set was examined with multiple response trajectory analysis, a method that has not been previously used to study co-occurrence of these symptoms.

**Results:**

We identified a five trajectory solution as the best: “very low” (19%), “low” (31%), “high” (22%), “late sharply increasing” (16%) and a “very high increasing” (12%). In the “late sharply increasing” and “very high increasing” groups the scores tended to increase with age, while in the other groups the levels were more stable. Overall, the results indicated that depressive, anxiety, and somatic symptoms co-exist from adolescence to midlife.

**Conclusions:**

The multiple response trajectory analysis confirmed high stability in the co-occurrence of depressive, anxiety, and somatic symptoms from adolescence to midlife. Clinicians should consider these findings to detect symptoms in their earliest phase in order to prevent the development of co-occurring high levels of symptoms.

**Electronic supplementary material:**

The online version of this article (10.1186/s12888-019-2203-7) contains supplementary material, which is available to authorized users.

## Introduction

Mental disorders, such as depression and anxiety, are a notable global health challenge and major contributors to the overall burden of disease, disability-adjusted life years, and suicide [1, 2]. Moreover, depressive and anxiety symptoms tend to co-exist with somatic symptoms. However, research on the co-occurrence and co-development of the symptoms is sparse, while the majority of studies focus only on depression [3]. To arrive at a more in-depth understanding of the joint co-occurrence of mental and somatic symptoms over time, studies focusing on trajectories of these different symptoms over the lifespan are needed.

The importance of further investigation of co-occurrence of symptoms was emphasized by the Zurich cohort study [4], which showed that stable comorbid states are more common than individual non-comorbid states. In other words, there is a clear comorbidity of anxiety or depression, which is stable from youth to adulthood. The strength of the relationships warrants further research to focus on the co-occurrence in particular, extending prior evidence about single disorders. Also, a review concludes that there are strong associations between depression, anxiety, and somatic symptoms, and suggests a reconsideration of existing nosology and re-conceptualization of symptomatic relationships [5]. However, studies have relatively short follow-ups, and do not cover the lifespan from adolescence to midlife. The review further emphasized the need for population-based longitudinal studies focusing on the co-occurrence of depressive, anxiety, and somatic symptoms.

Among longitudinal studies examining individual symptoms, a myriad of studies have focused on depression or depressive symptoms under various conditions [6–14], whereas corresponding studies on anxiety [15–18], and in particular on somatic symptoms [19], are scarce. A couple of studies have investigated comorbid depressiveness-anxiousness [4, 20–23]. Overall, however, the focus areas or coverages of those previous studies are limited to either early adolescence, early childhood, older ages, or to one gender only, and the interest has been in finding possible correlations with various predictors rather than the co-occurrence of the symptoms and their course across the lifespan. Previous studies have further used different methods, e.g. log-linear models [4], whereas multiple response trajectory analyses [24–26] have not been previously used to examine the joint occurrence of the internalized symptoms.

Therefore, studying the joint trajectories in these symptoms, covering a notable part of lifespan and the periods when the symptoms often first occur, are warranted. If the midlife mental and psychosomatic disorders have their roots in earlier life, early detection of high-risk groups is needed. Moreover, if the symptom trajectories are stable over time, studies that aim to address and prevent these symptoms have to more strongly tackle the symptoms already in adolescence. A long follow-up from adolescence to midlife, and identifying common and unique joint trajectories in the symptoms, could help produce new information for more timely intervention. Moreover, the results obtained with this methodological approach could be taken into account when re-thinking diagnostic categories of mental health problems in future [5].

Thus, to fill in these gaps in previous evidence, the aim of this study was to apply multiple response trajectory analyses in order to provide novel evidence about the development and co-occurrence of depressive, anxiety, and somatic symptoms during the lifespan from adolescence to midlife. More specifically, we examined how far the three symptom types occur jointly, or whether specific trajectories where there are one type of symptoms only, could be identified. This is done by joint group-based trajectory analysis (GBTA) [24–26], a novel person-oriented method that enables identification of development of the three symptoms types simultaneously. This kind of methodology has not been previously applied in the field of mental health.

## Methods

### Participants

Data for this analysis are derived from a longitudinal study, which was set up in a medium-sized industrial city of Luleå in Northern Sweden [27]. All students in their last year of compulsory education in every school in that region participated at baseline in 1981 (506 girls and 577 boys). Practically all of the originally invited students consented to participate in the baseline investigation, i.e., 1080 out of 1083 boys and girls (99.7%). Participants have been followed up in altogether four waves: in 1981 (baseline age 16 years), 1986 (age 21 years), 1995 (age 30 years), and 2008 (age 43 years) with an exceptionally high response rate over the follow-up (more than 90% throughout the waves, Table 1). There was also a follow-up at age 18, but mental health variables could not be constructed at age 18, as the items in 1983 questionnaire were partly different. Otherwise, the format of the data collection was kept similar at all time points.

**Table 1:** Numbers of participants and their percentage of the original population at each study wave

	Men (numbers)	% of original	Women (numbers)	% of original
Original	577		506	
16 years	574	99.5	506	100
21 years	560	97.1	500	98.8
30 years	551	95.5	495	97.8
43 years	523	90.6	487	96.2

The baseline survey at age 16 was completed in classrooms, while the follow-up surveys were collected in classmate re-unions, by mail or by telephone using trained professionals. Telephone interviews were made mainly for those with reading and writing difficulties, and some of the late responders. However, mainly, surveys were based on self-rate paper format questionnaires. Two reminders were sent out. The response rates were very high, so the reminders needed to only be sent to a small number of those, who were not present when the surveys were filled in. Thus, at age 16 almost everyone filled in the questionnaire during school hours. At the follow-ups after compulsory school, all cohort participants were invited to class reunions. Those who could not come received the questionnaire by post, followed by reminders by post or by phone.

The interviewees were asked extensive sets of questions about their health. Additionally socio-economic and clinical information was collected and measured [28]. Final data used for this analysis thus comprised 1,001 participants (482 women and 519 men).

 The study was approved by the Regional Ethical Review Board in Umeå, Sweden. Response to a survey was considered as consent to participate in the study. Moreover, information about the study was given orally to all pupils before the study began. In addition, all pupils and their parents received written information about the study including that it was voluntary to participate. During all years, it was stated in the questionnaire that participation was voluntary and that they could leave the study at any time without any explanation.

### Measures

Mental health was measured following previous procedures [27, 28]. However, to obtain more distinctive variables for the trajectory analyses each item was included only in one of the scores. Moreover, ‘backache or sciatica’ was not included in the somatic symptoms score as the conditions it measures in midlife were considered to differ too much from those in teenage [29].

Anxiety symptoms score comprised four items: 1. restlessness, 2. concentration difficulties, 3. worry or anxiety, and 4. anxiety or panic. Those who responded “No” to all four symptoms scored 0. Another question asked about frequency of the symptom(s). Those who responded “Off and on” or “Never”, and had one or more of the symptoms, scored 1, while those responding “Often” or “All the time” scored 2. Total score was computed as the mean of the four items, ranging continuously from 0 to 2.

We included five symptoms for the depressive symptoms score: 1. sleeping problems (0–3), 2. poor appetite (0–2), 3. general tiredness (0–2), 4. felt down and sad (0–3), and 5. dejected about future (0–3). Items with response range from 0 to 3 were recoded to 0–2 by combining the intermediate responses. The score was computed as the mean of the five included items.

Somatic symptoms score was based on six out of 43 symptoms: 1. headache or migraine, 2. nausea, 3. breathlessness, 4. dizziness, 5. overstrain, and 6. palpitations or stomach problems. These symptoms were selected to represent, according to an expert panel (64–96% agreed), ‘functional’ or ‘psychosomatic’ symptoms during adolescence and early adulthood, and development of the prevalence of these symptoms over time [29]. All items scored from 0 to 2, and the total score was computed as the mean of the six included individual item scores.

### Statistical analysis

Individual changes in mental health symptoms over time were investigated by a multivariate version of group-based trajectory analysis (GBTA) [24–26]. The model used here estimates joint developmental trajectories of three distinct symptoms: depressive, anxiety, and somatic symptoms.

The mixture normal distribution was applied to depressive and somatic symptoms, but the variable anxiety was considered under mixture Poisson distribution. The shape of the developmental progression curve for each symptom was defined using statistical considerations. Depressive symptoms score, for example, may not develop linearly with age. Therefore, a linear shape would be insufficient and hence a second degree polynomial curve and the so-called broken stick model (knot point age 21 [29]) was tested for each of the variables. Note that a third degree polynomial model is not feasible in our situation, since it yields to fully saturated model (4 measurement points, 4 parameters to be estimated). It turned out that for depressive and somatic symptoms, the broken-stick model was better than the second degree curve, but for anxiety symptoms, the second degree model had a better fit. The modeling was done in several steps using the statistical software R with the package “flexmix” [30]. The determination of the number of trajectory groups and their shapes (linear, curvilinear, broken-stick) was based on visual inspection, plots of posterior probabilities (rootograms) and Bayesian Information Criterion (BIC) [30]. Men and women were analyzed together, but additional sensitivity analyses were done stratified by gender due to possible known differences in the level and incidence of the symptoms between women and men [5].

The preferred number of groups is based on the use of BIC information criterion. Here we tested the number of groups k = 1,…,8. The BIC-values were: BIC(k = 1) = 15095.559; BIC(k = 2) = 10937.163; BIC(k = 3) = 10118.299; BIC(k = 4) = 9811.781; BIC(k = 5) = 9541.862; BIC(k = 6) = 9411.699; BIC(k = 7) = 9329.122 and BIC(k = 8) = 9284.083. It turned out that BIC values do not converge to the clear minimum point, but the degrease in values for k = 6, 7 and 8 is only marginal (see also the plot in the Additional file 1: Figure S1). Therefore, we used the solution of the five groups k = 5, where neither group becomes too small to be practical. Furthermore, the choice of five groups is also well supported by reasonable practical interpretations and by the U-shaped rootograms (see the Additional file 2: Figure S2). Finally, missing data were very small, and were removed at the modeling stage.

## Results

Mental health and somatic symptoms of the entire cohort by wave (age at baseline and at each included follow-up point) are described in Table 2. Anxiety and somatic symptoms appeared to increase with age, while depressive symptoms did not depend on age in any systematic manner. However, mean depressive symptoms were higher at the beginning of the follow-up as compared to the final follow-up point.

**Table 2:** Means and standard deviations (SD) of depressive, anxiety and somatic symptoms at baseline (16 years) and at the follow-up waves, and the total number of participants for men and women

	Depressive symptoms	Anxiety symptoms	Somatic symptoms	Total number of participants
	Mean (SD)	Mean (SD)	Mean (SD)	
16 years	0.54 (0.32)	0.13 (0.26)	0.28 (0.26)	*n* = 996
21 years	0.45 (0.31)	0.16 (0.34)	0.24 (0.25)	n = 996
30 years	0.50 (0.33)	0.22 (0.45)	0.35 (0.31)	*n* = 981
43 years	0.49 (0.37)	0.23 (0.43)	0.37 (0.34)	n = 996

**Fig. 1:**
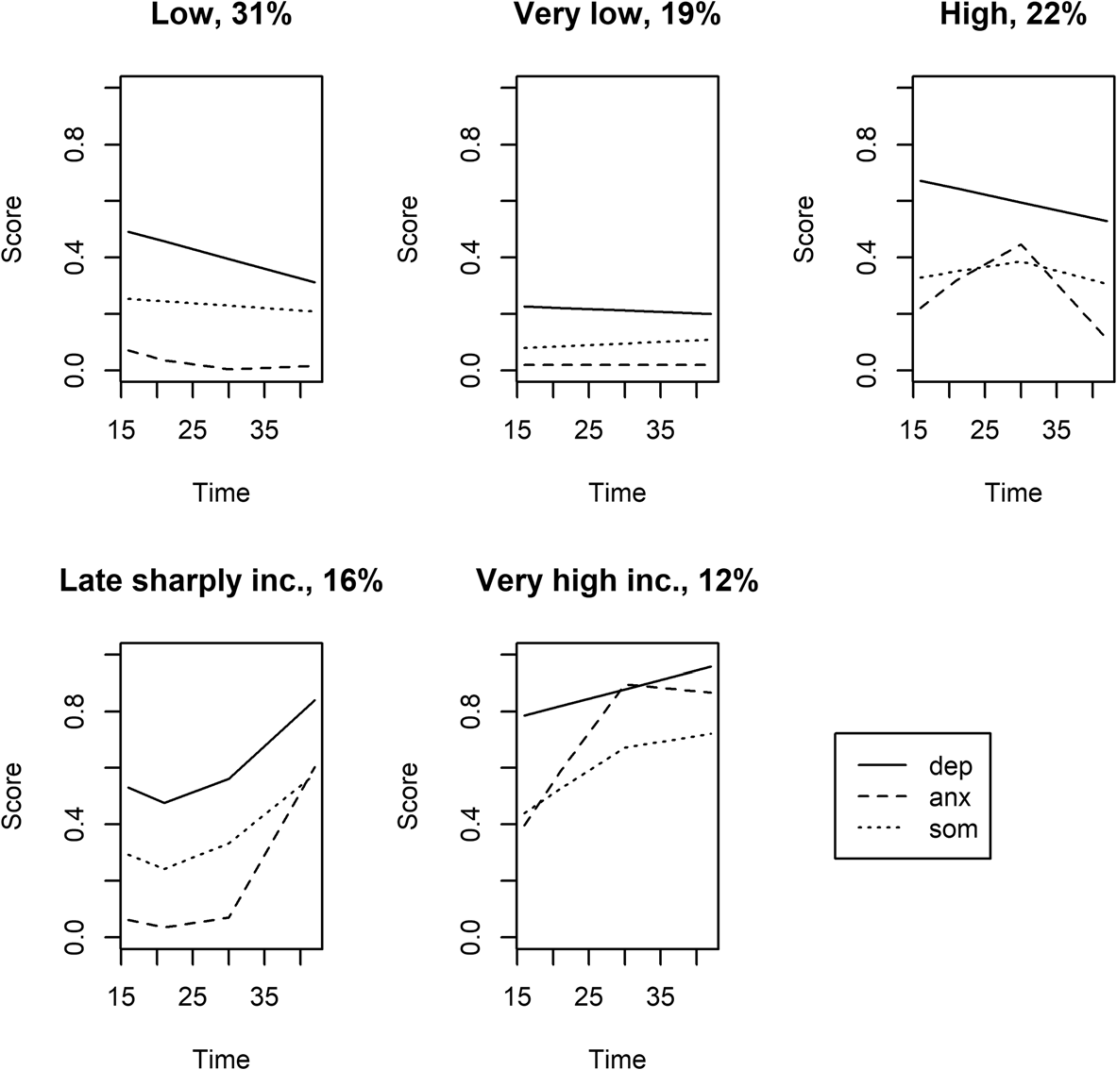
Multivariate trajectory plots of depressive, anxiety, and somatic symptoms scores in men and women from age 16 to age 43. [Late sharply inc. (late sharply increasing), Very high inc. (very high increasing)]

The groups identified by the joint trajectory analyses are described in Fig. 1 by means of the scores in each time point. The analysis discerned five distinctive groups: “very low” (19%), “low” (31%), “high” (22%), “late sharply increasing” (16%) and a “very high increasing” (12%). In the first mentioned three groups, all three scores tended to decrease by age, whereas in the “late sharply increasing” and “very high increasing” groups all curves sloped clearly upwards. Overall, the analysis indicates that depressive, anxiety, and somatic symptoms co-exist from adolescence to midlife in a stable manner, or that the level at one point during the lifespan is strongly associated with the past level, and also with the future level of all types of symptoms.

## Discussion

This is the first study to examine the joint co-occurrence of depressive, anxiety and somatic symptoms over 27 years, covering the critical periods of transition e.g. first from adolescence to adulthood, then education, transition into paid employment, and working life span until midlife. Joint trajectory analysis identified quite a consistent co-occurring trajectory sets in this sample, reflecting 5 distinct groups of very high and high levels of all three symptom types, late sharply increasing, and more average-to-low level trajectory sets.

### Interpretation

Previous studies suggest that depressive, anxiety, and somatic symptoms are correlated [5]. However, to our knowledge, no previous study has combined the cross-sectional and longitudinal approaches as has been done in this present study. By virtue of the multiple response trajectory analysis, this study adds to previous knowledge about the joint occurrence of the symptoms, highlighting the stability of their co-occurrence also in the long run during the lifespan.

More specifically, examining co-occurrence of different symptoms from adolescence to midlife, this study sought to identify what the similarities and differences are in the development of these symptoms and their co-occurrence. Furthermore, we focused on the different shapes and different directions in the curves. As our cohort is a random population sample, the results can also be interpreted to depict average development of the included symptoms by age. It should be noted that a “normal” development and course of these symptoms during the lifespan is not assumed to be zero-level. However, the joint group-based trajectory analyses break down the average into several groups with different symptom levels. The size of these groups helps us to understand the normal development.

The “very high increasing ” trajectory group appeared to be characterized by increasing symptoms, in addition to the high levels. The steepest increase was seen in anxiety symptoms, the increase was evident in somatic symptoms, and also visible in depressive symptoms. The high level of the symptoms co-occurring during a long period in the life span is a novel finding of this study. This clear co-occurrence should be considered in future studies and when addressing any of these symptoms in any interventions or health promotion/disease prevention programs. Moreover, also practicing clinicians facing people with e.g. high levels of depressive symptoms, might need to aim to identify the presence and levels of the other symptoms as well. Furthermore, these results suggest that clinicians should endeavor to detect symptoms in their earliest possible phase, to prevent the development of co-occurring high levels of depressive, anxiety, and somatic symptoms to continue, or to increase over time. Thus, while the measured symptoms per se are not often clinically relevant, taken together the three measures could have clinical relevance, suggesting that screening for symptoms might warrant referral to clinical care. Nonetheless, as this is an epidemiological study, it needs to be acknowledged that it is not possible to draw any *direct* conclusions or implications for clinical practice. Successful prevention would, however, have important public health and societal significance and implications, as trajectories of mental disorders are associated with poor labor market outcomes such as unemployment and being neither in education, employment, nor training [31, 32].

Additionally, when further interpreting our results, one might consider the concept of comorbidity. We focused on the symptoms of two key affective mental health disorders, anxiety and depressive symptoms. However, our data do not enable further confirmation of these patterns or factors behind potentially varying patterns. Moreover, as we focused on symptoms, and not medical conditions, we preferred to refer to co-occurrence of the symptoms rather than comorbidity. Although high levels of the included symptoms can be “morbid”, the results of this study are highlighted from the public health and societal perspective, particularly emphasizing the joint trajectories of the included symptoms. Their potential consequences could be assumed to be related to the stability of work careers, for example [31, 32].

The results further showed that while depressive and anxiety symptoms co-occurred, depressive symptoms increased for the groups with late sharply increasing and very high and increasing level of symptoms, but tended to slightly decrease for the other groups from adolescence to adulthood. In turn, anxiety symptoms tended to be either stable low or very low, or increase first and decrease or be stable later, or increase only after the age of 30 years. The rationale for focusing on depressive and anxiety symptoms together was to confirm whether the increase in anxiety could be observed in both symptoms. In all, the results suggest that when being on a trajectory of high levels of depressive symptoms or increasing depressive symptoms, it is most likely that the person is also on a high-level or high and increasing trajectories of anxiety and somatic symptoms. This means that symptoms do not typically change from depressive symptoms to anxiety or the other way round, but the co-occurrence of these symptoms appears to be a long-term situation over the lifespan. Such an interpretation is also line with the evidence that mental health problems begin early in life and high levels of symptoms are persistent from childhood to adulthood [31–33].

As early-onset mental disorders are a key cause of work disability [34, 35], an implication of this study is that interventions for their prevention and treatment should be targeted as early as possible. This is based on our findings regarding the high stability of the symptoms, and high and increasing level of co-occurring symptoms already from adolescence. Thus, any interventions in later midlife are likely to be less effective. In another words, as the co-occurrence of the high level of symptoms has its roots in adolescence, preventive measures need to be targeted as early as possible, before the symptoms develop. Given that the burden of mental disorders is even projected to grow in the future, this further highlights the need for early detection of the risk factors of mental ill-health before young people become economically inactive [36–38].

### Methodological considerations

A limitation of this study is the relatively small sample size, which did not allow for a more detailed sub-group analysis. It needs to be further acknowledged that we examined symptoms instead of clinical depression, anxiety disorder, or somatic diseases. However, similar symptoms are typically collected in surveys. The validation of the variables used has been published earlier in more detail, and the measures have been found to have acceptable face validity [27, 28]. Thus, although the self-reported symptoms are not to be used for any diagnosis, most of the same questions are included e.g. in DSM V diagnostic criteria for depression and anxiety [39]. Somatic symptoms have, in turn, been found to predict severe mental disorders [40], although we acknowledge that the measure is complex and under debate [41]. Furthermore, we cannot exclude that some of the symptoms were due to severe diseases, such as lung disease development, for example. However, as the participants were adolescents at baseline, and relatively young at the end of the follow-up as well, severe somatic causes are less likely than e.g. in elderly populations as a cause of the symptoms. Nonetheless, we preferred to use the term somatic symptoms in place of functional somatic symptoms for better accuracy. Moreover, we acknowledge that our somatic symptoms do not make an actual somatic symptom disorder, which would necessitate inclusion of some critical aspects such as excessive thoughts, feelings, or behaviors related to the somatic symptoms [39]. As our main interest was not specifically on the somatic symptoms or somatic symptoms disorder, but on the joint occurrence of somatic and mental symptoms, missing some items is less crucial. Although we used key symptoms of somatic symptoms, as judged by experts and following previous procedures [28], some of the symptoms further are more common than others. Thus, the score does not distinguish between the symptoms, which could partly account for the results. However, the sample size did not allow for more detailed groupings of the symptoms, nor for their severity. One might further have high levels of some somatic symptoms and low levels of mental symptoms, but co-occurrence still appears strong. Thus, during the follow-up of 27 years, participants showed either low/medium or high/increasing levels in all the symptoms, and the analytical method did not find groups constituting exceptions to the co-occurrence shown. Instead, the levels remained stable throughout the follow-up.

The strengths of our study were a long follow-up and the opportunity to examine paths from adolescence to midlife, and the joint trajectory modeling, providing novel evidence about mental health trajectories and co-occurrence of symptoms during the lifespan. Furthermore, both women and men were included, and showed similar development over time despite known difference in the prevalence of mental and somatic symptoms [5, 29]. Also in our additional analyses (data not shown), women were more likely to belong to the high level trajectories and men to low level trajectories. However, joint development of symptoms was similar, and the same trajectory groups were identified in the gender stratified analyses, justifying our decision to show the results pooling women and men. Additionally, response rates to the surveys remained exceptionally high throughout the follow-up, with 94% of still living participants responding at the final survey [27]. Thus, the cohort remained representative of the target population, and non-response and attrition are unlikely to account for findings to any great extent, such as changes in mental health. Among the small percentage of non-respondents, boys were somewhat overrepresented, as well as those with low grades from their compulsory education, and boys and girls with low parental education. We acknowledge that it remains possible that many contextual factors influenced the response pattern, as is always the case when collecting any data. While only one area in Sweden was included, the cohort has been shown to be representative of the same-age Swedish population in relation to sociodemographic factors, health status, and health behavior [27]. Finally, a particular strength of this study was the use of a method, namely a Group Based Trajectory Analysis (GBTA) that can be seen as a novel contribution to this area. More specifically, with the GBTA we were able to produce novel evidence about the development of the co-occurrence of depressive, anxiety and somatic symptoms from adolescence to midlife. Advantages of GBTA are the ability to identify qualitatively distinct developmental curves that are not directly observable from the series of repeated measurements of mean development. Thus, GTBA represented an alternative to the more traditional longitudinal analyses, where the analysis is most often based on the assumption of a single homogenous population. Thus, groups were obtained from the data, without any prior assumptions about the groups. With the method, results can be obtained and visualized either in an easily understandable format or in a broader statistical detail. Moreover, when the method is compared to e.g. latent growth mixture modeling, GBTA provides a more parsimonious multivariate model due to simple variance-covariance structure. In the more flexible latent growth mixture model with different covariance parameters for all the variables the number of estimated parameters easily grows too large for practical multivariate implementations. Thus, GBTA provides the opportunity to identify groups of people in the data that have similar development in their symptoms over time. In other words, this a more person-oriented approach, where we do not make strong assumptions beforehand, or classify people based on any predefined cut points to specific groups. Instead, the GBTA allowed us to identify what kind of actual latent groups there are in the data. It should be noted though that as GBTA is based on mixture models and groups are only approximations, careful interpretation of the results is important, and using them accordingly.

In conclusion, the results of this study suggest that co-occurrence of high and increasing levels of depressive, anxiety and somatic symptoms can be seen in the joint trajectories across the lifespan, from adolescence to midlife. These findings should be considered in clinical practice to detect mental health problems in their early phase, and paying special attention to the potential co-occurring symptoms. In addition to better maintenance of mental health, this is likely to have further significant societal impacts, e.g. in the prevention of work disability and other adverse employment outcomes, supporting current efforts to extend work careers also from their beginning and in early midlife.

## Additional files

Additional file 1: **Figure S1.** The plot of the model selection criteria for the selection of the best number of trajectory groups: the Akaike Information Criterion (AIC), Bayesian Information Criterion (BIC) and Integrated Complete Likelihood (ICL). (TIF 91 kb)

Additional file 2: **Figure S2.** Rootograms of posterior probabilities for the model selection for the selected number of trajectory groups. (TIF 198 kb)

## Data Availability

The datasets used and/or analyzed during the current study are available from the authors on reasonable request.

## Abbreviations

BIC: Bayesian Information Criterion
GBTA: Group-based trajectory analysis
SD: Standard deviationsss
