# Supplementary figures and images for: Co-occurrence of depressive, anxiety, and somatic symptoms: trajectories from adolescence to midlife using group-based joint trajectory analysis
Source: BMC Psychiatry. 2019 Aug 1;19:236. doi: 10.1186/s12888-019-2203-7 (PMC6670180; doi:10.1186/s12888-019-2203-7)

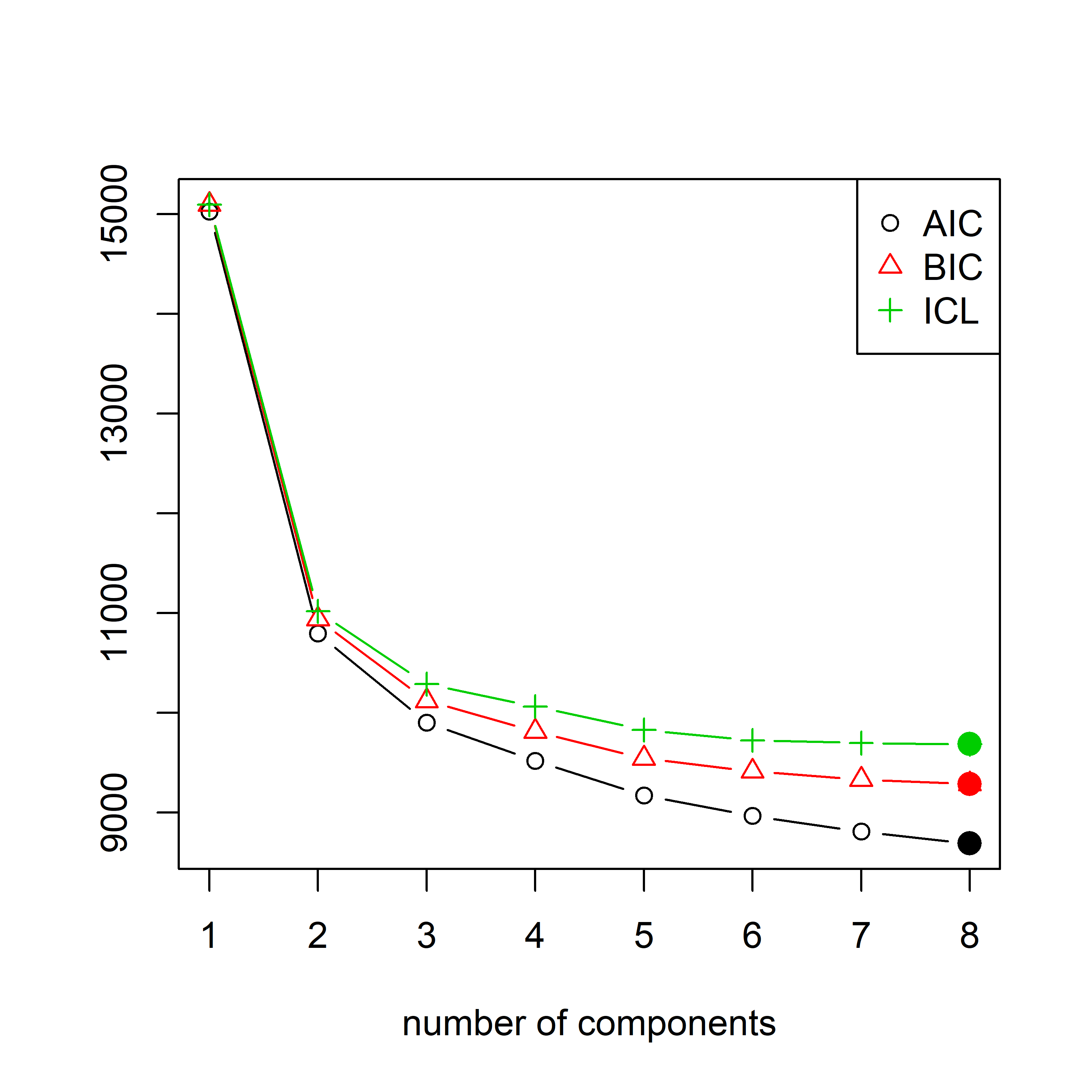

Supplement: Supplementary file 1 — Figure S1. The plot of the model selection criteria for the selection of the best number of trajectory groups: the Akaike Information Criterion (AIC), Bayesian Information Criterion (BIC) and Integrated Complete Likelihood (ICL). (TIF 91 kb) [file 12888_2019_2203_MOESM1_ESM.tif]

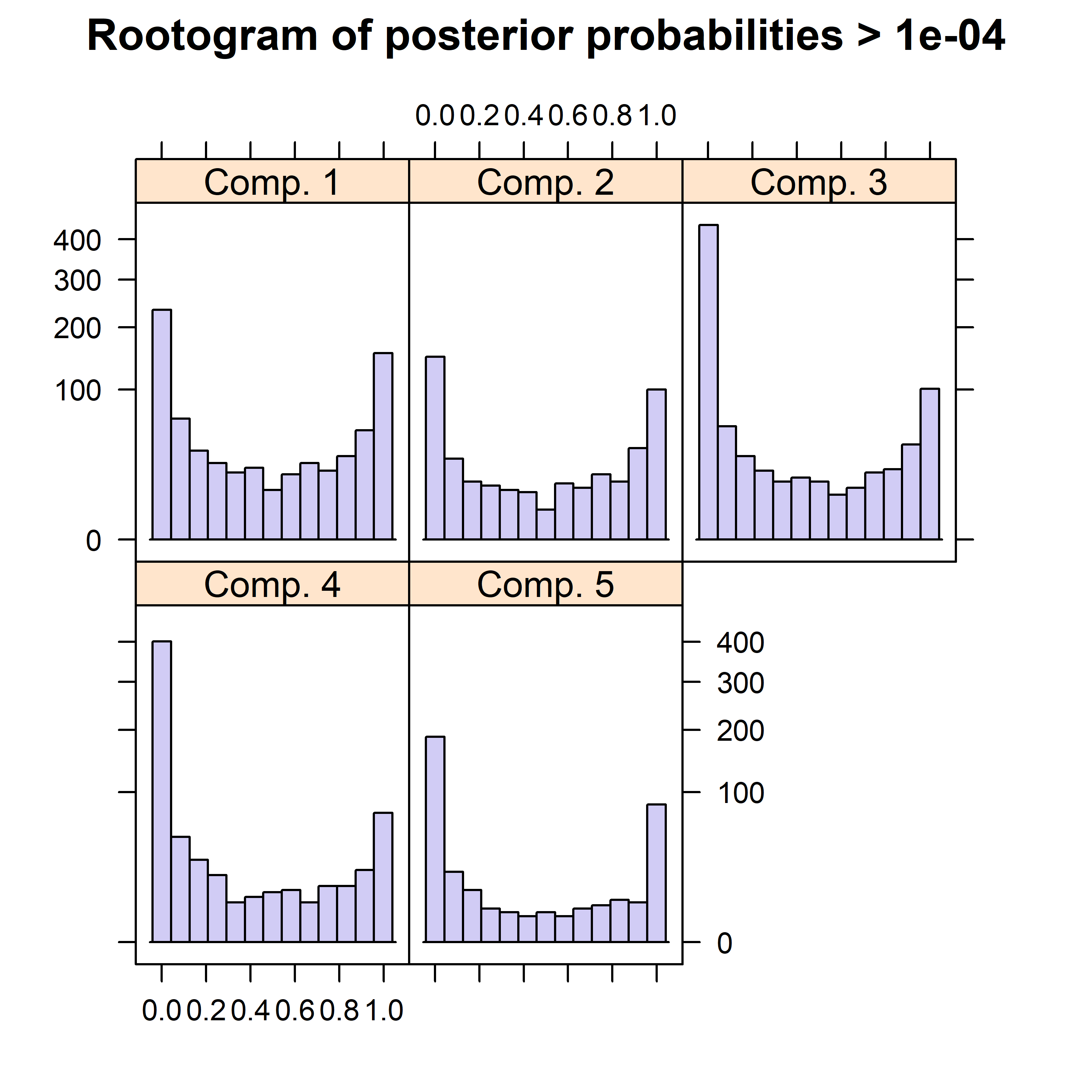

Supplement: Supplementary file 2 — Figure S2. Rootograms of posterior probabilities for the model selection for the selected number of trajectory groups. (TIF 198 kb) [file 12888_2019_2203_MOESM2_ESM.tif]
